# Lund University Cardiac Arrest System and Percutaneous Coronary Intervention During Cardiac Arrest: Case Report and Review of Literature

**DOI:** 10.7759/cureus.21159

**Published:** 2022-01-12

**Authors:** Arsh N Patel, Chao-Wei Hwang

**Affiliations:** 1 Department of Research, Alabama College of Osteopathic Medicine, Dothan, USA; 2 Division of Cardiology, Johns Hopkins University School of Medicine, Baltimore, USA

**Keywords:** cardiac arrest, acute st-elevation myocardial infarction, st-elevation myocardial infarction (stemi), lucas device, ventricular fibrillation, in hospital cardiac arrest, primary percutaneous coronary intervention (pci), lucas assisted pci

## Abstract

We present a case of a 45-year-old female who presented to a community hospital with an anterior ST-elevation myocardial infarction (STEMI) that subsequently developed prolonged ventricular fibrillation (VF) refractory to repeated defibrillation and antiarrhythmic medications. Primary percutaneous coronary intervention was performed in the patient with VF but supported only by the Lund University Cardiac Arrest System (LUCAS). Despite a total VF time of 127 minutes, the patient was eventually discharged neurologically intact with a normal left ventricular function. For the right patient, this case illustrates the utility of the LUCAS device, especially at community hospitals without immediate venoarterial extracorporeal membrane oxygenation or ventricular assist device capability.

## Introduction

We present a remarkable case of a middle-aged female who presented to a community hospital with acute myocardial infarction and developed ventricular fibrillation that persisted for 127 minutes despite advanced cardiac life support and emergency percutaneous coronary intervention. She was supported by the Lund University Cardiac Arrest System (LUCAS) device and remained mostly conscious during her prolonged cardiac arrest, subsequently making a full recovery after a long hospital course. The LUCAS device was a key in achieving return of spontaneous circulation (ROSC) and maintaining neurologic function for this patient. Despite the high mortality rates of patients in cardiac arrest, there is some evidence to support the benefit of utilizing the LUCAS device to perform effective high-quality chest compressions to achieve positive patient outcomes [1].

## Case presentation

**Figure 1:**
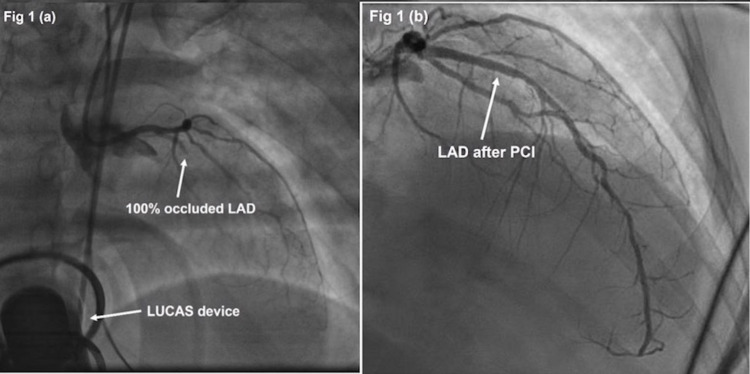
Proximal Left Anterior Descending Artery Occlusion (a) Occlusion of the proximal left anterior descending artery (LAD) with Thrombolysis in Myocardial Infarction (TIMI) flow grade 0 pre-percutaneous coronary intervention (PCI). Lund University Cardiac Arrest System (LUCAS) device visible in the background. (b) Restoration with TIMI flow grade 3 after deploying two drug-eluting stents in the LAD.

A 45-year-old female with hypertension, hyperlipidemia, and tobacco habituation presented with an hour of crushing substernal chest pain. ECG showed ST-segment elevations in leads V2, V3, and V4 with ST-segment depressions in leads II, III, and augmented vector foot (aVF) consistent with acute anterior ST-elevation myocardial infarction (STEMI). She was brought to the emergency department at a community hospital, where she developed cardiac arrest with ventricular fibrillation (VF) shortly after arrival. The patient was intubated, and the LUCAS device was applied. The patient remained in refractory VF despite receiving 30 shocks, a 300 mg amiodarone bolus and infusion, and a 100 mg lidocaine bolus. She was in profound cardiogenic shock. She received 32 rounds of epinephrine, a norepinephrine infusion, a dopamine infusion, and multiple pushes of sodium bicarbonate. Fortunately, the LUCAS provided such effective chest compressions that despite being in continuous VF, she remained mostly conscious and responsive during her ordeal. Return of spontaneous circulation (ROSC) still could not be achieved after 66 minutes of continuous advanced cardiac life support and chest compressions in the emergency department. As the patient remained mostly conscious and followed simple commands even without ROSC, she was taken to the cardiac catheterization laboratory with VF and full cardiac arrest, supported by LUCAS chest compressions. She did receive midazolam and fentanyl for sedation and pain. In the catheterization lab, she underwent successful percutaneous coronary intervention (PCI) of a 100% thrombotically occluded proximal left anterior descending (LAD) artery with overlapping 3.5 x 15 mm and 3.0 x 23 mm drug-eluting stents (Figures 1a, 1b). She finally achieved ROSC a few minutes after reperfusion. She had been in continuous VF for 127 minutes in total. 

An intra-aortic balloon pump was placed, and the patient was airlifted to a tertiary center, where she was placed on venoarterial extracorporeal membrane oxygenation (ECMO). She had a stormy course in the intensive care unit (ICU), suffering acute respiratory distress syndrome, supratentorial and infratentorial intracranial hemorrhages, ischemic colitis complicated by gastrointestinal bleeding, vascular access site bleeding with retroperitoneal hemorrhage, and acute renal failure. However, she slowly began to make progress on all fronts. She was successfully decannulated from ECMO on hospital day seven. Miraculously, while she did suffer bouts of ICU delirium, her neurologic function remained intact. She was downgraded to a stepdown unit on hospital day 14. Her echocardiogram showed a preserved left ventricular ejection fraction of 50%-55%. She was eventually discharged home on hospital day 23. She only had vague recollections of the events that occurred during the day of the cardiac arrest. Her mood remained bright; she stopped smoking, enrolled in cardiac rehabilitation program soon after discharge, and very quickly became their “star patient.”

## Discussion

There is increased realization that manual chest compressions are frequently ineffective due to inconsistent compression rates, fatigue, and frequent interruption of compressions. The prognosis for cardiac arrest patients remains dismal, with some studies showing that only 5% survive till hospital discharge neurologically intact [2].

**Figure 2:**
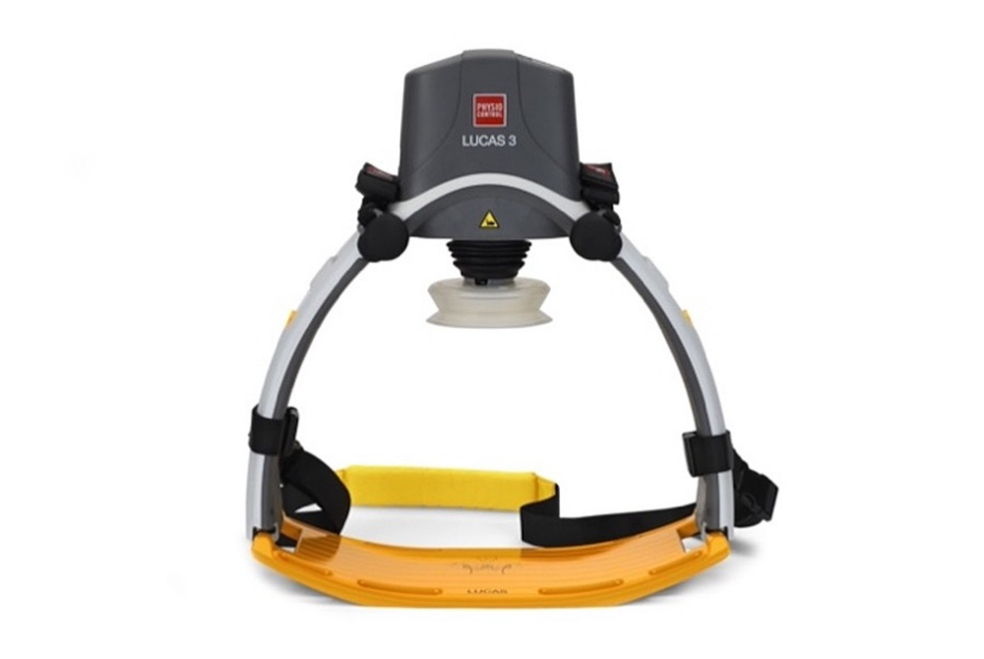
Lund University Cardiac Arrest System (LUCAS) Standard LUCAS 3 chest compression system produced by Stryker (Kalamazoo, Michigan, USA) used in the event of cardiac arrest.

The Lund University Cardiac Arrest System (LUCAS) is a device (Figure 2) that provides automated mechanical chest compressions for patients in cardiac arrest, with the hope that improved coronary and cerebral perfusion due to more effective and consistent chest compressions could lead to improved outcomes [3]. In the MECCA study (including 1,274 patients) [4], a prospective randomized study comparing LUCAS application in the ambulance to LUCAS application in the emergency room and to manual chest compressions in out-of-hospital cardiac arrests (OHCA), there was higher survival if the LUCAS device is applied early, in the ambulance, and within five minutes of cardiac arrest. In MECCA, the adjusted odds ratio for survival effect was 1.47 (p=0.026), favoring early LUCAS application compared to manual chest compressions [4]. Perhaps not surprisingly, outcomes are analogous for in-hospital cardiac arrest (IHCA) when using mechanical chest compressions. In a meta-analysis of nine studies comparing mechanical chest compression devices with manual chest compressions in adults with IHCA, Couper et al. found a significant overall improvement in survival rate to hospital discharge or to 30 days when using mechanical chest compressions [5]. A key factor in reaching optimal survival rates in cardiac arrest patients revolves around the timing of LUCAS application. In a single-center retrospective observational study of IHCA (containing 68 patients) in the emergency department, early LUCAS application led to higher four-hour survival rates compared to LUCAS application with a delay of four minutes or more (p<0.05) [6]. While statistical significance was not met owing to the small sample size, numerically more patients in the early LUCAS group achieved ROSC and survived admission [6]. 

Studies examining LUCAS use during PCI, as in this case report, are far more limited. There are currently no randomized trials involving LUCAS-assisted PCI. In a retrospective study, Almalla et al. evaluated the outcomes of patients with OHCA who underwent LUCAS-assisted PCI [1]. Of 219 OHCA patients in the study, 56 patients were brought to the catheterization lab without achieving ROSC for LUCAS-assisted PCI, and only one patient survived till hospital discharge. Outcomes for LUCAS-assisted PCI for cardiac arrest occurring in the catheterization lab seem to be less dire. For this patient population, Wagner et al. found that 11 of 43 patients in their small study survived till hospital discharge with normal neurologic status [1,7]. Despite the limited trials and studies, there are data supporting that for the right patient, LUCAS-assisted PCI can provide a means of survival.

## Conclusions

This case report followed a 45-year-old female with prolonged ventricular fibrillation who made a remarkable recovery after LUCAS-assisted PCI. The limited data that are available suggest that the LUCAS may be beneficial to achieve ROSC. LUCAS application, especially early, may play a pivotal role in improving outcomes for both IHCA and OHCA demographics. As this case illustrates, LUCAS-assisted PCI offers a path to survival for the right patient, especially in community hospitals without on-site ECMO or ventricular assist device capability.
